# Core Principles and Practices for the Design, Implementation, and Evaluation of Social and Behavior Change for Nutrition in Low- and Middle-Income Contexts with Special Applications for Nutrition-Sensitive Agriculture

**DOI:** 10.1016/j.cdnut.2024.104414

**Published:** 2024-07-14

**Authors:** Mary Packard-Winkler, Lenette Golding, Tsedenia Tewodros, Emily Faerber, Amy Webb Girard

**Affiliations:** 1 Independent Consultant, Bethesda, Maryland, United States; 2 Save the Children U.S., Department of Global Health, Washington, DC, United States; 3 Hubert Department of Global Health, Rollins School of Public Health at Emory University, Atlanta, GA, United States; 4 Dietetics and Nutrition Department, College of Health, University of Alaska Anchorage, Anchorage, AK, United States; 5 Nutrition and Health Sciences Program, Laney Graduate School, Emory University, Atlanta, GA, United States

**Keywords:** low- and middle-income countries, maternal, infant and young child nutrition, nutrition-sensitive agriculture projects, project implementation, social and behavior change

## Abstract

**Background:**

There is currently no cogent set of standards to guide the design, implementation and evaluation of nutrition social and behavior change (SBC), including for nutrition-sensitive agriculture (NSA).

**Objectives:**

We aimed to capture, consolidate, and describe SBC core principles and practices (CPPs), reflecting professional consensus, and to offer programmatic examples that illustrate their application for NSA projects in low- and middle-income countries.

**Methods:**

We conducted a narrative review following a 4-step iterative process to identify and describe SBC CPPs. We first reviewed general SBC frameworks and technical documents and developed a preliminary list of CPPs and their definitions. Following review and feedback from 8 content experts, we revised the CPPs, incorporating the panel’s feedback, and conducted a more specific search of the peer-reviewed and gray literature. We presented a revised draft of the CPPs to 26 NSA researchers, practitioners, and implementers at the 2022 Agriculture, Nutrition and Health Academy annual conference. We then conducted a focused review of each CPP, and 3 content experts rereviewed the final draft.

**Results:**

We reviewed ∼475 documents and resources resulting in a set of 4 core principles: *1*) following a systematic, strategic method in designing, implementing, and evaluating SBC activities; *2*) ensuring design and implementation are evidence-based; *3*) grounding design and implementation in theory; and *4*) authentically engaging communities. Additionally, we identified 11 core practices and mapped these to the different stages in the SBC design, implementation, and evaluation cycle. Detailed descriptions, illustrative examples and resources for implementation are provided for each CPP.

**Conclusions:**

An explicit set of CPPs for SBC can serve as a guide for design, research, implementation, and evaluation of nutrition and NSA programs; help standardize knowledge sharing and production; and contribute to improved quality of implementation. Broader consultation with SBC practitioners and researchers will further consensus on this work.

## Introduction

Human dietary behavior is complex. Not only must decisions on what and how to eat be made multiple times each day, a host of complementary and often competing factors influence each procurement, preparation, and eating episode. These influencers range from internal determinants such as personal habits, values, preferences, agency, and self-efficacy to broader social and structural constraints such as time, availability and accessibility of nutritious food, discriminatory social norms, gender-based barriers, and religious obligations. Nutrition-sensitive agriculture (NSA) seeks to improve diets and nutrition by addressing underlying determinants of suboptimal intakes of nutritious food [1]. Existing frameworks and conceptual models highlight 3 key pathways through which NSA interventions may increase consumption of nutritious foods, particularly among women and children [[2], [3], [4]]. These pathways focus on *1*) increased production of nutritious foods to improve accessibility and availability for consumption; *2*) increased purchasing power through strategies that enhance agriculture incomes, improve agricultural markets, or policy changes that alter price points; and *3*) women’s agency and empowerment through enhanced income, decision making, time allocation, and health.

Recent systematic reviews confirm the ability of NSA interventions to improve women’s and children’s diets in low- and middle-income countries (LMICs), though impacts are often variable and less than anticipated [[4], [5], [6], [7]]. Research in the field of NSA documents greater effectiveness when interventions include nutrition social and behavior change (SBC) activities to shift diet practices [8,9]. Nutrition SBC interventions seek to identify and shape influencing factors and foster healthier diet patterns. However, in an updated review of NSA, Ruel et al. [5] note that the SBC component of NSA programs is a “common bottleneck in implementation” and highlight the need to identify “best practices in the design and implementation of effective and affordable” SBC for NSA. Similar calls have been noted for the broader field of maternal and child nutrition in LMICs [[10], [11], [12], [13], [14], [15], [16], [17]]. Moreover, while we have a consensus on professional standards and practice guidelines in clinical nutrition as well as public health nutrition more broadly [[18], [19], [20]], the more specific field of nutrition SBC, including NSA, has not articulated agreement on what implementation factors contribute to successful dietary behavior change and maintenance. Despite these gaps, evidence on “what works” in the broader field of nutrition SBC is growing [10,14,16,17,21,22]. This has fueled the development of a range of guidance documents and training packages for implementers working in SBC more broadly as well as in the field of nutrition [[23], [24], [25], [26], [27], [28]], including for NSA [29]. This body of literature suggests a consensus on core principles and practices for nutrition SBC generally, which also apply to NSA programming.

This narrative review aims to capture, consolidate, articulate, and justify those core principles and practices and their application in the field of nutrition SBC and, where possible, for NSA more specifically. In doing so, we bring together existing literature and experience related to SBC implementation to:

• Describe the core principles and practices clearly and concisely, especially for practitioners.
• Offer examples to illustrate those principles and practices, particularly emphasizing applications for NSA projects.
• Provide practical resources on the implementation of core practices, when available.

This narrative review articulates our working understanding of SBC core practices and principles that can be applied to any SBC initiative, although our focus is on the implementation of SBC in the context of nutrition and NSA. We conceptualize *core principles* as the overarching, cross-cutting elements forming the foundation for implementing quality SBC programming. *Core practices* are the key actions SBC practitioners undertake throughout all project cycle stages to ensure effective implementation. We use the term *project* to describe a discrete activity or set of activities “implemented by a defined set of implementers and designed to achieve specific objectives within specified resources and implementation schedules” [30], while we use the term *activity* to describe “a specific action or process undertaken over a specific period of time by an organization to convert resources to products or services to achieve results” [30]. For example, many NSA projects have a variety of SBC activities all working toward food production and consumption behavior changes related to the program aims. We use the term “core” to avoid the judgmental connotations of the word “best” and to convey more precisely the fundamental, key practices that practitioners have increasingly coalesced around as being critical to effective SBC implementation.

## Methods

Our motivation to conduct this narrative review of SBC core principles and practices arose as part of a global landscaping of SBC implementation designed to improve diet quality in the context of NSA projects in LMICs. This landscaping study includes 2 core components. The first is a systematic review of SBC activities in the context of NSA to document commonly used strategies, approaches, and techniques and how these influence project effectiveness (Tewodros, T, Escobar, C. X., Berra, L. S., Girard, A. W). The second component engaged ongoing NSA projects to understand the design, implementation, and evaluation of their SBC activities in real time. It included document review, interviews, and site visits of selected NSA projects in LMICs with in-depth reviews of the SBC strategies aimed at changing diet practices to improve diet quality. In planning and implementing the analysis of our data for these 2 aims, we sought recognized standards against which to benchmark analyses. Although we did not identify such a set of standards, we did identify a growing consensus on what constitutes core principles and practices in SBC more broadly—with application to the field of nutrition in LMICs and NSA more specifically—and a need for their consolidation. Therefore, we sought to fill this gap by conducting a narrative review of SBC with the goal of identifying, compiling, and describing these core principles and practices along with illustrative examples and online implementation resources into an easily accessible resource [31].

**FIGURE 1:**
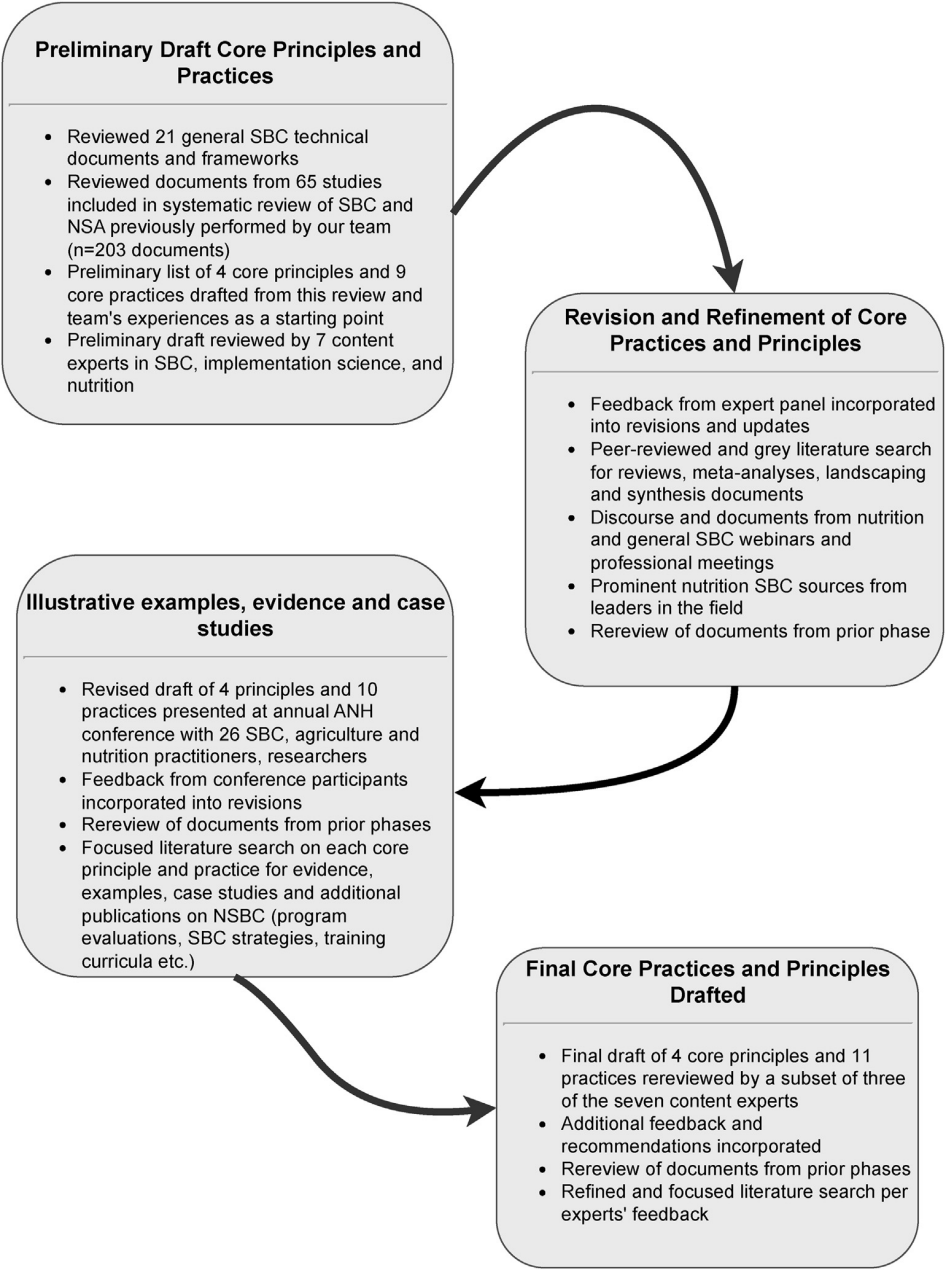
Steps in the narrative review process for the development of core principles and practices in nutrition social and behavior change.

To achieve this aim, our team followed an iterative, 4-step process (Figure 1) to document, describe and identify core principles and practices along with representative examples emerging from research and practice to date. Our team first reviewed existing general SBC technical documents and frameworks related to the systematic design and implementation process for SBC more broadly (*n* = 21; Supplemental Table 1). From this review of technical documents and frameworks, we identified principles and practices that were commonly put forward as guidance for what SBC implementers should do to optimize behavioral outcomes. From these general documents and our review of the studies identified for the aforementioned systematic review of SBC in NSA (*n* = 65 studies and 205 documents), we then developed a preliminary list and description of 4 core principles and 9 practices reflecting broad consensus, including an explanation of how each core practice was broadly defined.

Seven content experts with expertise in nutrition, implementation science, NSA, and SBC reviewed this initial draft and provided extensive feedback. Content experts came from donor, nongovernmental organization (NGO), and research institutes and represented the United States, Europe, South Asia, and Sub-Saharan Africa. All held advanced degrees (Masters’ or Doctorate) and >15 y of research and/or programming experience in nutrition or SBC. Several also had cross-cutting expertise in SBC related to gender or agriculture. Based on their feedback, we then conducted a more detailed search of peer-reviewed and gray literature with an emphasis on reviews and synthesis documents related to SBC and nutrition. For this second stage of the narrative review, we searched Academic Search Premier, PubMed, and Google Scholar for peer-reviewed narrative reviews, systematic reviews, meta-analyses, and expert commentary on SBC to improve maternal, infant, and young child nutrition in LMICs. This search utilized broad search terms including “behavior change” AND “nutrition” OR “nutrition sensitive agriculture” and prioritized documents focused on maternal and child nutrition or NSA in LMICs and published prior to 1 January, 2024. We also similarly identified and reviewed gray literature related to SBC, nutrition, and NSA including guidelines, frameworks, and reviews. Gray literature was identified through internet searches of donor and technical support websites and platforms, research institute databases such as the International Food Policy Research Institute, United Nations agencies, and international NGOs using search terms such as “behavior change” AND “nutrition” OR “nutrition sensitive agriculture.” Gray literature also included discourse and documents from webinars and professional meetings focused on SBC as well as prominent SBC resources from several nutrition-related SBC and other integrated SBC projects in LMICs. (Illustrative examples include and are not limited to Alive & Thrive, Breakthrough ACTION & RESEARCH; C-Change, Deutsche Gesellschaft für Internationale Zusammenarbeit [GIZ] GmbH; United States Agency for International Development [USAID] Advancing Nutrition program; USAID Strengthening Partnerships, Results, and Innovations in Nutrition Globally [SPRING] program.) We employed snowballing to identify additional relevant citations and resources from these documents as well as from review of citation tracking tools in PubMed and Google Scholar (e.g., reverse snowballing, [32]). Throughout this review process, we documented how others defined and organized key elements of SBC processes, principles, and practices; we noted consistent trends and commonalities, and eventually discarded “outliers” to focus on areas of consensus. Through this second round of iterative review, we elaborated and refined the list of core principles, practices and their descriptions, including the addition of 1 new core practice related to evaluation.

This revised draft of core principles, practices and descriptions was then presented for review and comment to a cohort of 26 SBC, nutrition, and agriculture practitioners and researchers at a side session of the 2022 Agriculture, Nutrition and Health Academy Conference [33]. Participants represented academic, nongovernmental, and governmental agencies working on NSA in LMICs in Africa and Asia. Feedback from this session provided further revisions and refinements to the descriptions of the core practices and principles, including the addition of 1 new core practice related to applying formative research to design, and identified the need for illustrative examples and case studies.

For our third round of iterative review, we undertook a more focused literature search to identify evidence, examples, resources, and case studies for each of the core principles and practices. This final stage involved *1*) identification of peer-reviewed literature in PubMed using broad search terms relevant to each core principle or practice (e.g., “formative research” and “nutrition”); *2*) a review of the studies included in the aforementioned systematic review of SBC in NSA; and *3*) snowballing and reverse snowballing of resources identified in prior rounds of review [32]. We additionally drew on our prior search of the gray literature to identify nutrition and NSA SBC projects and did additional internet searching using the relevant NGO, donor, agency, or research institute database as well as common search engines (e.g., BING, GOOGLE), for project-related materials such as formative research documents, SBC strategies, training manuals, curriculum, and monitoring and evaluation reports. For project-based and case study searches, we limited document review to those focused on maternal and child nutrition or NSA in LMIC contexts and that included SBC as an explicit intervention strategy. An exception to these criteria relates to searches for methodology-based core practices (e.g., monitoring/evaluation). The revised and elaborated core principles and practices, supporting evidence, and case studies were then rereviewed by 3 of the content experts who provided initial review with additional feedback incorporated as indicated.

Overall, our search strategy for this narrative review followed a highly iterative and interpretivist approach. Throughout the development and subsequent revisionary stages of this narrative review, our team continuously discussed emerging findings and feedback received from our content experts and reviewers. As new information, concepts and ideas emerged from these discussions and feedback, we iteratively identified new search terms and refined existing ones. We continuously returned to the literature to identify additional supporting or refuting evidence, examples, case studies, or resources as per feedback from content experts, reviewers, and our team-based discussions. Due to this iterative team-based process, we were unable to continually track with high accuracy the total number of unique articles, documents, and resources reviewed. However, our team estimates we reviewed ∼475 articles, documents, examples, and resources, with ∼310 included in the main text and supplemental materials. Of these, 240 contributed to a core principle or practice and are included in Supplemental Table 2.

## Findings

Our review identified 4 core principles and 11 core practices. We first describe the core principles followed by a review of each of the core practices.

### Core principles

#### Systematic and strategic

The first core principle of quality SBC is to follow a systematic, strategic process in the design, implementation, monitoring, and evaluation of SBC activities [16,34]. A systematic and strategic approach is based on a methodical rationale; it includes an explicit statement of aims, and a logical articulation of what, how, and why each element of the SBC strategy was chosen, how they relate to each other, and how they will contribute to achieving stated behavior change aims.

In 1982, the Johns Hopkins University Center for Communication Programs developed the “P Process,” the first framework designed to guide practitioners as they plan strategic, evidence-based health communication programs [35]. The P Process added rigor to SBC communication programs. Since then, a basic 5-step process for SBC has been adopted, adapted, and promoted by various public health and nutrition organizations (Supplemental Table 3 [23,27,[35], [36], [37]]). More recently, FHI360 produced an Adaptive Management Framework Toolkit that outlines the SBC process applying an adaptive management lens to the design, implementation, monitoring, evaluation, and adaptation of SBC interventions [38].

The basic 5-step process is summarized below:

• **Understand:** Conduct primary research and use secondary research to understand the situation, context, and drivers of key behaviors.
• **Design, test, and adapt:** Design the SBC strategy, including the approach, materials, and tools, using formative research and existing evidence. Pilot test and refine elements with community participation before larger-scale implementation.
• **Implement, monitor, and adapt:** Deliver SBC activities with fidelity. Systematically collect data on SBC activity implementation to track progress, learn what is working, and where there are gaps. Apply learnings and adapt to strengthen efficiency and effectiveness. Update the SBC strategy annually, based on learning and the evolving theory of change (TOC).
• **Evaluate, learn, and adapt:** Measure outcomes, assess impact, disseminate results, determine future needs, and revise your strategy.

Although the steps may suggest a linear process, SBC implementation is an iterative process. Implementing teams should embrace movement back and forth between steps, as ongoing learning and adaptation is a key to success. In each stage of the process, a commitment to continual learning fuels adaptation and refinement of the program’s focus and methods, enhancing effectiveness. In more complex and dynamic multisectorial projects, the need for rapid, participatory, and adaptive learning processes that can support continuous implementation improvements are even more critical [[39], [40], [41]]. Development of a TOC and strategy (based on evidence and social and behavioral theory, principles that are discussed next) is a core part of making an SBC initiative strategic and systematic. Recent reviews highlight the need for interventions to provide sufficiently detailed descriptions of the process, theory, and evidence base used to develop a TOC and strategy and its underlying assumptions [11,14,[42], [43], [44], [45]]. Such detail fosters transparency, allows for replication, and gives greater insights into what specific techniques work to change behavior and why [46]. By providing the rationale for how and why a set of SBC activities will lead to the desired behavior change, a TOC serves as a process map for implementation and can guide the development of a sound monitoring and evaluation strategy [47]. A TOC differs from a logic model in that the TOC specifies underlying assumptions and includes causal mechanisms to show why each intervention component is expected to result in the intended outcomes; in other words, it is explanatory (e.g., Jacob Arriola et al. [48] and Cole et al. [49]). A TOC should be considered a living tool, to be reviewed and updated regularly, based on project learning [47,50]. In contrast, a logic model is descriptive, depicting the intervention’s intended outcomes. A program impact pathway (e.g., [21]), also used in program planning and evaluation, provides a more focused and practical roadmap for achieving program goals and assessing impact [46,51]. Developing a TOC will make developing the overarching SBC strategy more focused.

#### Evidence-based

The next core principle involves evidence-based design, implementation, and evaluation. Evidence-based practices make “decisions based on the best available scientific evidence, using data and information systems systematically…conducting sound evaluation, and disseminating what is learned” [52]. Various sources can supply the evidence base for a project’s design and ongoing implementation. For example, to justify a project’s goal, data may come from global research, or a national health database. At the same time, relevant assessments may shape its objectives. Selection of focal behaviors and SBC approaches will likely be based on the formative research that practitioners conduct in the local context to understand the situation, the people, and factors influencing behavior. Research, including outcome and impact evaluations, is an essential foundation of evidence generation. However, retrospectively knowing what worked in a particular project does not reliably answer the question of what works in general, because the dynamics of approaches and activities and their potential effectiveness varies in different contexts. A grounding in contextual data as well as relevant evidence associated with an approach or activity enhances the quality of its design, implementation, and evaluation of its effectiveness [16,19,45,53,54]. Furthermore, a commitment to evidence generation and ongoing learning throughout a project’s life cycle can inform adaptation and continual improvement for enhanced effectiveness throughout implementation.

A variety of research methods and data sources can be mobilized to build a project’s evidence base, ranging from desk reviews and situation analyses, pilot testing, field trials, and fidelity assessments, as well as routine monitoring of outputs and processes. Dissemination of learnings—not only from impact evaluations but including process data on adaptations, unexpected outcomes, and failures—is critical to advance the field’s understanding of the extent to which an approach or activity works, how it functions, for whom, and under what circumstances. We elaborate on this principle of using evidence in the core practices related to formative research, testing and adaptation, and monitoring and evaluation.

#### Theory-informed

The third core principle is to ground SBC project design, implementation, and evaluation in theories of behavior and behavior change from the social and behavioral sciences along with communication theory to enhance the potential effectiveness of approaches and SBC activities [[55], [56], [57], [58], [59]]. Theories of behavior and behavior change represent “the accumulated knowledge of the mechanisms of action (mediators) and moderators of change as well as the a priori assumptions about what human behavior is, and what the influences on it are” [55]. Communication theory refers to the body of theories that constitute our understanding of the communication process [60]. Applying theory provides more precise and nuanced explanations of why and how people behave the way they do [61]. Without a theoretical framework, any changes in behavior of individuals or populations will be difficult to explain [61]. Too often, SBC activities in the context of nutrition interventions are designed without reference to theory; NSA is no exception [10,12,17,[62], [63], [64], [65]]. Given this, we elaborate the importance of identification and application of theory in more detail here.

Theories from a wide range of disciplines have been developed to explain or predict human behavior, although no single theory dominates SBC, nor should it. The problems, behaviors, populations, culture, and contexts of nutrition practice are broad, varied, complex, and always situated in social and environmental contexts. Nevertheless, many theories applied to public health programming focus on individuals as the unit of change and typically emphasize individual capabilities and motivation rather than broader social and environmental variables [55]. Addressing an issue may require integrating multiple theories and models, and SBC practitioners should embrace an ecological perspective that considers interpersonal, organizational, and environmental factors in activities aimed at influencing behavior [66]. Indeed, theories are part of an iterative process of designing, implementing, and evaluating SBC [23]; they can be used in complementary combinations for different phases of implementation or behavioral determinants [23,43,[67], [68], [69], [70]]. For example, a project may start with the Health Belief Model (HBM) [71] to guide the formative research on nutrition behaviors, but if data indicate that structural barriers are making it difficult for people to practice healthy nutrition behaviors, that model could be integrated with others capturing the environmental context, such as the Social-Ecological Model (SEM). Meanwhile, the Capability, Opportunity, Motivation → Behavior model can help focus design on factors influencing behavior and selecting which of these components to address through interventions [34]. Theory can also guide evaluation, ensuring theoretically derived determinants (mechanisms of action) of behavior and associated SBC activity components are monitored and measured [55].

Evidence is mixed as to whether projects explicitly based on theory are more effective than those that are not [[72], [73], [74]]. Nevertheless, theory is often poorly applied [55]. Although many practitioners will claim a project’s SBC activities are theory-based, they often do so without demonstrating how a theory has been used to design, monitor or evaluate SBC activities [73,[75], [76], [77], [78]]. Another factor is the inappropriate selection of theories [55]. Too often, a popular theory (e.g., Theory of Planned Behavior, Social Learning Theory, SEM, HBM) is made to work, rather than selecting a theory based on a particular purpose (e.g., to design a mass communication campaign or to shift social norms).

Moreover, inconsistencies are evident within the behavioral and social sciences regarding definitions and interpretations of what constitutes a theory [61]. Often, theory is used interchangeably with several other terms (e.g., concept, framework, model, logic model, pathway, TOC [55,61]), which can lead to confusion among SBC practitioners. Recognizing these challenges, we refer readers to several helpful online tools and resources for selecting or applying theory. These include the web-based tool, Theory Picker [79], The Theoretical Domains Framework [[80], [81], [82]], and the United States National Cancer Institute’s Group Evaluated Measures, a web-based database that provides descriptions of theoretical constructs and behavioral and social measures to assess these constructs [83].

#### Community-engaged

Another core principle of SBC is to authentically engage community members throughout a project’s design, implementation, monitoring, and evaluation to achieve long-term, sustainable community impact [[84], [85], [86]]. Specifically, this means working *with* communities and enlisting their active participation in identifying and analyzing problems, setting priorities, planning and designing, implementing, monitoring and evaluating solutions, and mobilizing resources [87,88]. Community members such as politicians, faith and other community leaders, service providers, researchers, and other critical decision makers can provide key contextual considerations to the SBC strategy and its implementation and evaluation [89]. Creating and sustaining the relationships that support authentic community engagement is best done through dialogical techniques that promote discussion, joint decision making, and empowering action that shifts the seat of power from implementers to communities [88,90,91].

There is a growing interest in community engagement to support collaborative, multilevel, culturally situated community interventions aimed at creating sustainable community-level impact. In 2009, an interdisciplinary conference convening on community engagement research developed a new paradigm that situates health as “embedded in a community ecology” that includes the community’s history, culture, social networks, and resources. Understanding and working through the local community allows planners and implementers to draw on local resources and leverage community platforms to solve problems [85]. Thus, community engagement empowers social groups and networks, builds upon local strengths and capacities, and improves local participation, ownership, adaptation, and communication. Such engagement avoids an overreliance on individual-level interventions divorced from community realities and promises more significant impact and sustainability [66,85]. More recently, others have developed dialogical approaches to community engagement grounded in local culture and use participatory processes that prioritize local knowledge and decision-making processes for authentic community engagement [92,93]. For instance, in 2020, UNICEF described 16 minimum quality standards for community engagement that address 4 challenges in current practice (i.e., quality, accountability, harmonization, and optimization) and can be used to structure community engagement activities, among other things [88].

The literature indicates there is no one-size-fits-all approach to community engagement as it involves complex interactions, making it context-specific. A 2015 meta-analysis of 131 community-engaged projects in high-income countries, including 13 focused on obesity and weight loss, noted that community-engaged approaches facilitated enhanced outcomes and social support. However, no straightforward method of engagement emerged as more effective than others [94]. In a review of global NSA projects in LMIC contexts, Leite [95] observed that projects often demonstrate varying degrees of “community engagement” and that these differ by the stages of the project cycle, with most of the higher-level engagement occurring at the formative phase. However, those projects applying participatory styles of community engagement across more stages of the project cycle achieved more significant impacts on dietary outcomes.

### Core practices

We now shift our focus to the 11 identified core practices that apply at different stages in the SBC cycle.

#### Prioritize a limited number of behaviors

One of the most critical early steps in the SBC design process is the identification of a manageable number of evidence-based behaviors. This step is crucial to successful implementation, as it guides focused investment in resources. The process involves prioritizing and selecting behaviors, and this is where the real challenge lies. It requires excluding behaviors that may not significantly impact the project’s core objectives or are less amenable to change through the project’s core activities. For instance, when individuals and communities are asked to change too many behaviors, the chances of success are significantly diminished, leading to potential project failure [96]. This is due to limitations on individuals’ memory and the difficulty of focusing on many things simultaneously [97]. As well, resource constraints can limit frequency and reach if projects attempt to act on too many behaviors. This is why many SBC practitioners say, “Less Is More” and seek to prioritize behaviors based on research [26,28,98].

The first stage of an evidence-based process for the prioritization and selection of behaviors is typically based on a desk review of existing evidence. This stage helps to narrow the focus on behaviors that have demonstrated a positive impact on health, are likely to be feasible for the priority group to adopt, and are within the realm of influence for the program. Once this is completed, the pivotal role of formative research comes into play. Its findings provide a deeper, contextualized understanding, allowing a more refined focus on behaviors for the SBC design [28].

**FIGURE 2:**
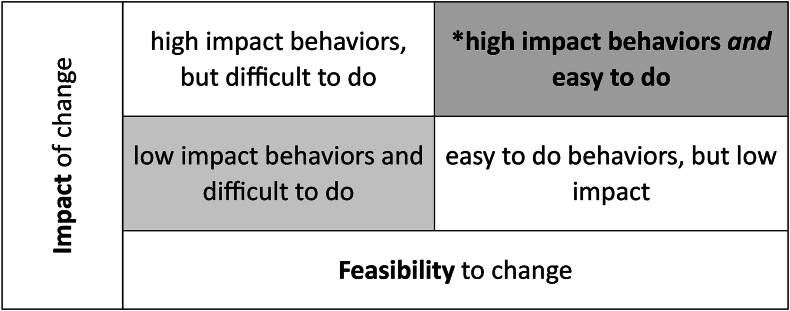
Feasibility and Impact grid to identify priority SBC behavioral outcomes [99].

It can be difficult for implementers to exclude behaviors that seem important. Guidance tools exist to help designers identify behaviors that have both a high potential impact on the project aims while also being feasible for the priority groups to practice. These include, for example, a prioritization guide from the United States Agency for International Development (USAID) Advancing Nutrition program [28] and a video developed by Alive & Thrive Ethiopia on small doable actions [99]. This video demonstrates how to select behaviors based on their potential impact and feasibility. It highlights the critical work of breaking complex behaviors into ‘small doable actions’ as part of the prioritization and focus process. Figure 2 [99] depicts a tool to guide a team’s work to brainstorm, identify and prioritize potential key behaviors. Although limited, growing evidence indicates the value of applying a prioritization process in SBC design [100,101]. For example, the Upscaling Participatory Action and Videos for Agriculture and Nutrition (UPAVAN) study exemplifies how a complex NSA project utilized evidence and theory to prioritize selected behaviors for SBC activities [100,102,103]. The project team identified multiple pathways for impact involving a range of specific behaviors, each associated with various possible factors. They narrowed their focus to a few behaviors based on consideration of the transtheoretical model of behavior change, conceptual frameworks, existing evidence, and formative research to learn what would be most relevant and feasible for the participants and local implementers [100]. After the team identified impactful and feasible behavior change pathways, specific behaviors were prioritized based on agronomic, economic, and social details uncovered in formative research and feasibility studies. For each key behavior, the implementers then considered various ways to increase capability, motivation, and opportunities for change.

#### Conduct research to understand context and behavioral influencers

Understanding the influential factors for a given behavior is critical to effectively shift that behavior [[104], [105], [106], [107], [108]]. The success of a nutrition SBC activity is heavily reliant on its alignment with the local context and its adaptability to the forces that steer dietary behaviors among intended participants. Formative research is a critical step in comprehending these forces, which encompass: environmental structures; sociocultural factors like cultural values, family systems, social norms, religion, and community resources; and individual factors such as skills, knowledge, habits, and beliefs. This research can identify the most influential forces, whether they act as barriers or enablers in shaping dietary behaviors. A thorough understanding of the local context is also essential for embedding activities in culture and fostering meaningful community engagement.

Various methods are useful for formative research, particularly in relation to projects intending to influence diets. A literature review of published and gray literature can bring understanding of the context and root causes of the problem and reveal knowledge gaps regarding key behaviors. Quantitative and qualitative approaches can then be used for primary data collection within the context and population of interest. Examples include focused ethnography [109,110], participatory assessments such as Participatory Rural Appraisal [111], Change Through Culture [112], Trials of Improved Practices (TIPs) [104,[113], [114], [115]], Doer/Non-Doer surveys [116] and Barrier Analysis [117], Human-Centered Design (HCD) [[118], [119], [120], [121]], and Positive Deviance analysis [[122], [123], [124]]. Taking a culture-centered approach to formative research and subsequent SBC design enables practitioners to explore the significance of food taboos and how meanings are culturally constructed through social interactions and structures to influence diets [110,125,126]. Knowledge, Attitude and Practices (KAP) surveys, a quantitative method with predefined questions in standardized questionnaires, can be used to discover potential barriers to behavior change. However, due to the limitations of survey data in capturing contextual information, it is advisable to combine methods like KAP studies with other more qualitative methods, such as those described above, and triangulate findings [127,128].

Newer linear programming methods such as Optifood [129,130] and market-based analyses such as Cost of the Diet [131] permit designers to develop diet pattern recommendations that are culturally grounded, locally available, and affordable. These methods also enable designers to model how agriculture, market or government subsidy interventions can shift availability and affordability of target foods. The application of multiple approaches to formative research permits triangulation and yields greater confidence in the findings and subsequent application to project design. Irrespective of the approach used, the research plan should be strategically designed to understand the myriad underlying motivators of the key behavior, fit a project’s budget and make the best use of existing sources.

#### Apply formative research findings to design

SBC strategies grounded in formative research and tailored to the key audience groups are more likely to drive meaningful and sustainable behavior change [28,108,[132], [133], [134], [135]]. Practitioners of effective SBC will analyze the data collected and use it to prioritize behaviors, develop a TOC, segment audiences, and decide what specific activities and approaches will best respond to the features of the local culture, social norms, experience, and behavioral drivers that have been identified. When projects define their messages and activities before research and data analysis have been done, they miss the opportunity to tailor SBC programming to fit the data and incorporate critical cultural and social insights that could significantly enhance the effectiveness of their interventions [136,137].

Another valuable application of research findings is in audience segmentation. This process is a crucial activity that divides a large, heterogenous audience into more homogenous groups of people (segments) with similar needs, values, or characteristics. The segmentation process recognizes that different groups respond differently to services, products, and communication messaging and permits the development of targeted communication strategies. Segmenting also enables a project to identify and focus on the most critical groups that influence change. A combination of qualitative and quantitative methods has the potential to provide more comprehensive insights to researchers in segmenting priority groups for SBC activities [138]. Quantitative methods can apply cluster analysis techniques post-hoc to create homogenous groups based on the participants’ answers. However, qualitative methods provide a more nuanced understanding of participant values, motives, and behaviors. When segmenting groups for SBC activities, it is helpful to focus on factors most likely to drive the intent to change behavior (e.g., needs, norms, self-efficacy) rather than just demographics or geography [[138], [139], [140]]. When planning approaches for different audiences, designers also should carefully consider the role of gender and other characteristics such as age, ethnicity, class, and social roles in influencing power structures and behavior change [16,[141], [142], [143], [144]].

Analyzing formative data and deciding how to apply it in project design can be a significant challenge for practitioners. Practical guidance includes Designing for Behavior Change [145], the Behavior Change Wheel [34], Intervention Mapping [57,146,147] and IDEO’s Human-Centered Design Field Guide [148]. USAID Advancing Nutrition has also developed 2 helpful technical briefs: Using Research to Design a Social and Behavior Change Strategy for Multi-Sectoral Nutrition [28] and an SBC Formative Research Decision Tree [149].

#### Build upon existing structures

Integration with ongoing structures in a project context can enhance local ownership and sustainability of SBC activities, rather than implementing isolated activities that end with a project’s closeout. A prominent example in global health links the delivery of vitamin A supplementation with vaccine campaigns. In NSA, working through agriculture extension workers is common [49,102]. Other platforms offering opportunities for nutrition or NSA integration include community-based savings and loans groups [150], early child development initiatives [48,151], school feeding programs [152], faith-based organizations [153], as well as public–private partnerships [[154], [155], [156]]. By utilizing a community-engaged approach and investing in existing community platforms, projects can effectively strengthen the capacity of local partners. Engaging with and investing in local systems allows practitioners to leverage expertise, assets, and influence within the community, leading to increased local ownership, agency, and collective action [[157], [158], [159]]. Furthermore, community-based workers and volunteers are often established and trusted in the community. Their engagement can foster more efficient, culturally appropriate, and effective social behavior change interventions, as compared to newly created roles or external actors [34].

However, integration with existing systems is not without challenges. Community health workers (CHWs) and other frontline workers (FLWs), often the backbone of nutrition programs, continually bear the brunt of task shifting efforts to reduce costs, improve efficiency, and expand community outreach [160,161]. Task shifting can increase job scope and complexity and add work, economic, and emotional burdens. These additional responsibilities have implications for productivity, work quality, burnout, and turnover. Programs that seek to utilize these FLWs to deliver intervention activities must carefully consider existing workloads, ensure sufficient supportive supervision and resources to fulfill responsibilities (e.g., transport), and advocate for fair and appropriate incentivization.

#### Engage multiple levels and groups

Nutrition-related behaviors result from the complex interplay of personal, sociocultural, and environmental factors that represent levels of influence conceptualized by the SEM. The SEM underscores the need for projects to engage at multiple levels in the socioecological system and address the various factors that operate across it [23,35,45,162,144]. The layers of influence on an individual range from family and peers to community groups and leaders, to the broader enabling environment, which includes laws, economic structures, and cultural systems. Deciding which levels of the socioecological system to target and which specific groups to engage should be an evidence-based process done in the early stages of project design and informed by formative research.

A disproportionate focus on individual-level programmatic solutions can be counterproductive. For example, we know that if activities only target individual mothers, even if their attitude or skills change, they may be unable to improve child feeding practices due to the influence of their family, broader social norms, or access to certain foods. Change may be relatively easy to promote at the individual level but challenging to sustain without changes in the social or policy space to create an enabling environment [66]. Targeting multiple levels in an intervention often requires integrating activities at the community level, such as community mobilization and capacity strengthening, with activities at the interpersonal level such as those that engage individuals, families, and peer groups, while simultaneously working at a more macro level through strategies like policy advocacy for structural change and culturally grounded methods to shift social norms [104,[163], [164], [165]].

To optimize value from working across systemic layers, increasing SBC research supports more family- or community-centric approaches [14,163,[166], [167], [168], [169], [170]]. These approaches focus on changing the behaviors of key influencers, for example, husbands and grandmothers, to create an enabling family environment for change; or engaging faith leaders to shift social norms [16,[141], [142], [143], [144]]. The growing recognition of the need to go beyond individual-level activities has also spurred efforts to influence systems and structures at higher levels of the socioecological model [66,144] through intersectoral strategies that create an enabling environment with potentially more far-reaching effects on multiple behaviors [171,172]. For instance, social mobilization and accountability measures can help mobilize resources, strengthen the health system, and increase political commitment [[173], [174], [175], [176]], while advocacy influences policy through coalition building and translating technical evidence into policy recommendations [177,178].

A 2-y NSA project in Burkina Faso that aimed to improve maternal and child diets and nutrition provides an example of engaging multiple groups and levels. It worked at the individual and interpersonal level by targeting diet and nutrition-related household behaviors and expanded opportunities in the enabling environment level through integrated strategies to address gender norms, production of nutritious crops, and household food security, reporting significant impacts on key nutrition indicators [[179], [180], [181]].

#### Use multiple complementary approaches

The term “approaches” here refers to the methods used to engage different priority groups and influencers (see Box 1 and Supplemental Table 4). For most behaviors, it is necessary to go beyond raising awareness to identify strategies that allow designers and implementers to authentically connect with communities and empower them to act [16,182]. SBC evaluation, research, and experience suggest that projects using multiple channels or approaches are generally more effective [16,43,173,[183], [184], [185], [186]], including a recent systematic review of NSA [9]. Combining different approaches in one initiative allows projects to tailor methods to fit the preferences of varying groups, address multiple behavioral determinants, and identify the most appropriate and relevant communication and engagement methods for a given community. Synergistically layering approaches also increases the density with which the audience receives key messaging, increasing the likelihood of individuals to act. These findings echo a common theme in the SBC literature: the most successful projects use a variety of methods and media. They go well beyond providing information to raise awareness by implementing approaches such as skill building, community dialog, edutainment, and ongoing support from peers or influencers.

BOX 1 **Social and Behavior Change Approaches**

*Enabling environment approaches*

• Advocacy
• Social mobilization
• Behavioral economics
• Capacity building
• Provision of material/inputs

*Community-based approaches*

• Community outreach
• Community engagement
• Community dialog
• Education
• Community mobilization

*Communication (BCC) approaches*

• Interpersonal communication (IPC)
• Entertainment education (“edutainment”)
• Social marketing
• Mass media
• Mid-media
• Small media
• Digital and social media

*Other approaches*

• Human-centered design (HCD)

Alt-text: BOX 1

The selection of approaches should be based on formative data on behavioral factors, including barriers and motivators for change, and participants’ values, norms, and preferences. For instance, Baker et al. [186] notes that core approaches required for achieving results in infant and young child feeding programs include interpersonal communication, community mobilization, mass media, and evidence-based policy dialog and advocacy.

Alive & Thrive’s work in Ethiopia found that only 16% of women who were exposed to one type of communication activity (i.e., home visits by health extension workers/volunteers, sermons from religious leaders, community meetings, media, or food demonstrations) fed a child an egg, compared to 50% of women who were exposed to ≥5 activities [187]. Additionally, the program found that participants who were engaged in the participatory activities of community mobilization and interpersonal counseling, as well as being exposed to mass media campaigns, demonstrated more significant improvements in spending and consumption of high-nutrient foods compared to those exposed to only mass media [183].

#### Apply participatory methods

Development practitioners have emphasized the importance of participation for years, recognizing that the chance for sustainable, positive outcomes is greater when community members are actively engaged in the learning and change processes—including formative research, design, and monitoring [[188], [189], [190], [191]]. Twenty-five years ago, professionals from communication and community development fields proposed a participatory model for social change highlighting these key elements: *1*) a “shift from persuasion and the transmission of information from outside technical experts to dialogue, debate and negotiation on issues that resonate with members of the community”; and *2*) a call to “go beyond individual behavior to social norms, policies, culture and the supporting environment” [192].

A wide range of studies have supported the consensus that interactive, participatory methods that engage individuals, groups, households, and communities actively are more effective for capturing the voices of key, vulnerable groups and supporting positive changes at multiple levels [16,87,92,[193], [194], [195], [196], [197]]. Participatory, interactive methods follow time-tested adult learning principles [190,198,199] and have been included in the guidance for nutrition projects to effectively support behavior change [200]. Key principles for applying participatory methods found across the body of research and experience cited here include:

• Seeking and mobilizing local knowledge and experience.
• Demonstrating a respectful attitude that values and empowers underserved populations.
• Engaging interested, affected, and relevant groups and individuals in analysis, planning, delivery, and evaluation of activities.
• Use of visual tools, interactive methods, and skills practice in capacity strengthening.

There are a wide variety of participatory methods that can effectively apply these principles as they engage community members and facilitate meaningful nutrition behavior change. Examples include community dialog approaches such as “Stories Without an Ending” [90], the Community Action Cycle [87,91,201], and the Action Media Methodology [[202], [203], [204]], which engage community members in a consultative, analytical process to design SBC materials grounded in their own perspectives. Additionally, the “Make Me a Change Agent” curriculum [205,206] applies adult learning principles and adapts them to local contexts to make activities relevant, engaging, and fun—for both FLWs and participants—using techniques such as games, drama, debate, and demonstrations.

Examples of applying participatory methods include the UPAVAN trial in India, described earlier, which demonstrated significantly improved maternal and child diets when a combination of interactive methods were used, compared to controls without participatory methods [100,103,207]. Examples of participatory methods included nutrition-focused participatory learning and action cycles and discussion groups for NSA videos. Similarly, NSA interventions in Malawi and Tanzania applied a participatory agroecological approach aimed at reducing social inequity as well as participatory action research principles to engage communities on preferred agricultural activities, women’s empowerment, and dietary change. Monthly community dialog sessions, experiential learning activities, and theater served to engage community members of differing levels of influence [[208], [209], [210]].

#### Test and adapt tools, methods, and communication materials

Implementers can avoid costly mistakes and improve effectiveness by testing and adapting materials and activities before using them throughout the project. Testing can prevent dissemination of materials or use of methods that may be rejected by audiences and stakeholders. C-Change identifies 4 types of testing. “Each type serves different purposes and happens at different points during the materials development process” [24].

1. Concepts are the “big ideas” that underpin programming. **Concept testing** explores how ideas resonate with participants at an emotional and values level. It is done with simple, rough drafts, before resources are invested in fully developing materials. Concept testing is often done as part of or shortly following formative research, to ensure that materials are audience-centered. Unfortunately, this crucial step is often overlooked. For practitioners, The Compass group provides a step-by-step guide to concept testing on their website [211].
2. **A stakeholder review** by partners and gatekeepers occurs after materials have been drafted. It allows stakeholders to view draft tools, methods, and materials, provide key technical inputs and suggestions for revisions, and build consensus about their overall appropriateness [212].
3. After concept testing and stakeholder review, materials are pretested and adapted based on findings. **Pretesting** is critical to ensure effectiveness of SBC activities, as it systematically solicits feedback on how attractive, understandable, relevant, and appropriate a message or material is. Pretesting can be achieved through in-depth interviews, focus group discussions, or rapid listening groups comprised of a subsample of beneficiaries from a relevant target population [213]. Though typically applied for testing behaviors, a TIPs approach can also be applied to pretest materials and tools. For example, in Kenya [115], India [113], and Malawi [214], a TIPs approach was applied to pretest a Healthy Mother Healthy Baby Toolkit to improve diets in the first 1000 d and inform toolkit redesign, delivery platforms, and scale-up.
4. **Pilot testing**, also referred to as feasibility testing, involves implementing planned SBC activities and delivering materials and messages on a larger scale through different platforms to determine whether the materials are used effectively by their intended users in the project contexts [22]. Evaluation data are typically collected from priority audiences through observations, surveys, and focus group discussions. Beets et al. [215], identify key characteristics of pilot studies that can enhance assurances that an intervention is ready for larger scale testing or implementation. These include *1*) a sufficiently heterogeneous sample that reflects the target population sociocultural demographics and is appropriate for the complexity of the intervention; *2*) delivery during the pilot phase mimics that of the scaled version (e.g., which platforms are used, who delivers the intervention, levels of supervision, duration); *3*) the pilot study collects data on feasibility and preliminary effectiveness; and *4*) repiloting is conducted in the event major changes are made to the intervention design or implementation of the first pilot deviates substantially from the planned scaled-up version.

Before expending resources on developing any new materials, practitioners should review existing materials and evaluation results from other similar projects to learn how audiences responded to materials or activities and their impacts, if any. This prework allows design teams to identify which materials or activities can be used, adapted, what is missing, and the mistakes to avoid. Resources for this purpose can be collected from implementing organizations, government and technical partners, and online SBC resources such as Springboard, COMpass, and The Communication Initiative Network.

HCD is a participatory approach to generate ideas creatively and test prototypes in an iterative, cost effective fashion. Its principles can be applied to integrate partners more purposively in a co-creation process involving testing and development of materials and methods [121,216,217]. For example, in Kebbi State, Nigeria, Breakthrough ACTION and USAID Advancing Nutrition used an HCD approach to co-create with communities, tools to support CHWs’ compassionate counseling on nutrition [218]. In Burkina Faso, HCD was used to adapt a South African maternal nutrition mHealth video series to the local context [219].

#### Strengthen and maintain SBC competencies for quality implementation

Regardless of how well a project is designed and equipped with material resources, successful SBC outcomes are impacted by the competencies of staff and community-based change agents and the support they receive to perform their roles effectively. In addition to improving the quality of activities, strong SBC competencies are also crucial for sustainability. To enhance and maintain the competencies to deliver effective SBC, donors and practitioners must invest sufficient resources in systems to assess, train, monitor performance, and support continual quality improvement among staff, volunteers, and other change agents working on a project’s SBC activities.

Implementation research has shown that FLW performance is affected by financial incentives and role specificity; for example, the Alive & Thrive program found in Bangladesh that infant, women, and child feeding services were delivered with greater effectiveness by single-purpose, paid staff than community volunteers with multiple roles [13]. Reviews of USAID SBC programming noted common competency gaps, in particular, prioritizing key behaviors, conducting and applying appropriate formative research, using participatory methods (both in training SBC practitioners as well as delivering SBC activities), and monitoring the quality and effectiveness of specific interventions [220,221]. Core SBC competencies can be defined as “measurable, observable, and clearly defined knowledge, skills, and attitudes that are critical to job performance.” They generally fall into the categories of *1*) foundational knowledge, skills, and attitudes; *2*) SBC design and planning; *3*) implementation; and *4*) monitoring and evaluating SBC activities [222,223]. In addition to their role-specific skills and relevant training in the intervention’s agricultural focus, all team members involved with NSA SBC should have basic technical knowledge of nutrition, maternal, newborn, and child health, and water, sanitation, and hygiene (WASH). Equally important is that team members acquire an understanding of basic principles of SBC and how to apply them within the scope of their roles and responsibilities. The SBC advisor or manager will additionally need specialized skills for applying the results of research and designing SBC strategies, as well as mentoring and coaching skills for strengthening SBC competencies, and management skills to oversee research, training, and monitoring [205,[223], [224], [225], [226]].

Community-based change agents also require deep familiarity with the sociocultural context in which the project operates, along with strong interpersonal and group facilitation skills [206,224,227]. To apply participatory methods as described earlier, change agents should be able to engage community members in dialog, reflection, problem solving, goal setting, and action planning. To do this requires strong interpersonal communication skills based on deep listening and the ability to create ways for everyone to be equally heard, respected, and meaningfully engaged.

**FIGURE 3:**
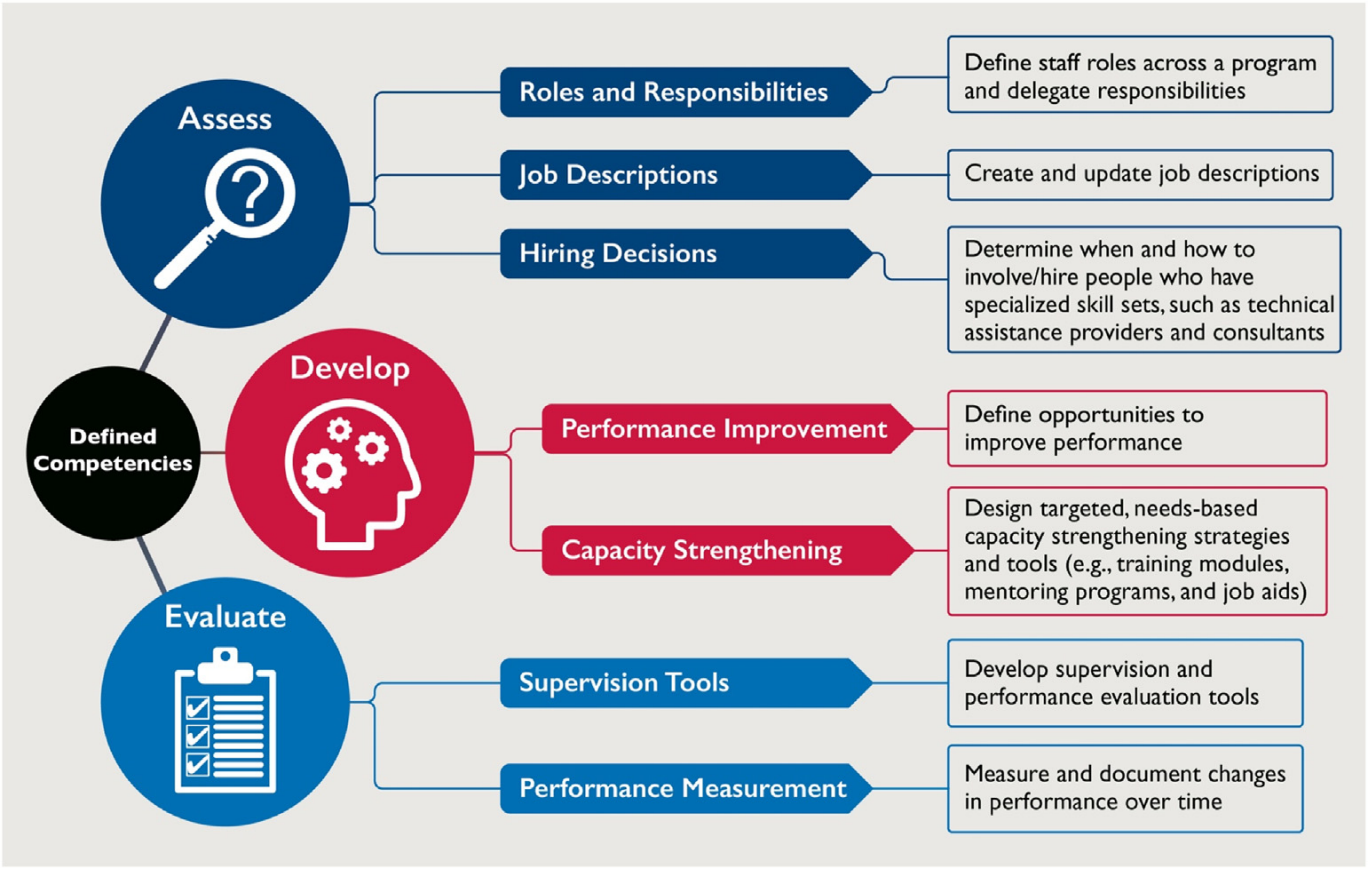
Elements of Social and Behavior Change Capacity Strengthening, from [222].

Strengthening the SBC competency of implementers may require changes in *their* behavior, and applying the same principles we use for SBC activities, namely: listening and learning from project staff and change agents, building upon their existing strengths, mobilizing their ideas for improvement, and using multiple methods to actively engage them in a participatory capacity-strengthening process [190,205,[228], [229], [230]]. The first step to ensuring workers can deliver quality SBC is to clearly articulate—and prioritize—the specific competencies needed [223,231]. Projects can then develop systems to assess, strengthen, and evaluate those competencies, as shown in the USAID Advancing Nutrition Schematic in Figure 3 [222]. Useful capacity strengthening toolkits are listed in Box 2 [205,206,222,223,225,226,[231], [232], [233], [234]]. The guidance presented in these tools highlights key principles for developing and maintaining the capacity of SBC practitioners:

• Provide skills-based training using participatory methods aligned with principles of adult learning [228].
• Follow up with refresher training, ongoing coaching and mentoring, and peer learning experiences to foster continual improvement.
• Monitor performance based on clearly defined core competencies that are customized for each activity and reflect evidence-based standards.
• Provide and train change agents to utilize user-friendly job aids that help them deliver activities effectively.
• Incorporate capacity strengthening into existing activities involving peer-based learning and periodic reflection meetings [223,231].

BOX 2 **Social and Behavior Change Capacity Strengthening Resources**

The Health Communication Capacity Collaborative – SBCC Capacity Ecosystem [226]

C-Change SBCC Capacity Assessment Tool [225]

Care Group Quality Improvement Verification Checklists [232,233]

Make Me a Change Agent [205,206]

Toolkits from USAID Advancing Nutrition and ASSIST programs [222,223,231,234]

Alt-text: BOX 2

#### Track implementation quality and measure progress

We recognize that while project outcomes may be fixed, the pathway to reaching those outcomes is not. Rather these pathways are often nonlinear, iterative, and sporadic. Implementing high-quality SBC requires a learning-focused and systematic approach to tracking and improving the quality of implementation along these dynamic pathways [38,39,235,236]. Monitoring, learning, and quality improvement are part of a project’s internal systems for ongoing learning and adaptation in such dynamic implementation contexts. Thus, this core practice includes monitoring implementation progress but goes beyond the traditional measuring of inputs, outputs, and outcomes. Instead, the focus is on the implementation processes that enable the team to rapidly recognize bottlenecks or problems, identify and implement solutions, and measure the impact of these changes on project implementation. Examples of such learning-focused systems include rapid cycle improvement methods [237,238], community quality improvement [[239], [240], [241]], and complexity-aware monitoring [[242], [243], [244], [245]]. These approaches emphasize “capturing and feeding back timely data” to permit “fine-tuning of the project, early insights on additional questions for analysis, and ongoing communication and the potential to learn from failure and success” [237].

There are several excellent resources for conducting quality assurance and quality improvement. Although most have been applied to the health sector [38,246,247], they are applicable to nutrition and NSA programming [[246], [247], [248]]. Although some quality assurance guides focus on the macro level, such as the Scaling Up Nutrition checklist for quality national nutrition plans [249], many are focused on implementation. They are generally based on an identified list of standards by which FLWs’ performance can be judged, measured, and enhanced, such as the Core Group’s Quality Checklist for Care Group Implementation [250]. Standards should be agreed-upon with stakeholders, donors, and practitioners and be based on effectiveness evidence. Given existing human and material resources, they should also be realistic for the practitioners. Then, tools to observe and measure performance against those standards can be employed. Project teams, ideally with representation from community members, then analyze results from quality monitoring, determine appropriate responses, and develop implementation and evaluation strategies. Projects that apply these approaches can more rapidly identify and address weaknesses in delivery, allowing them to prevent more significant problems down the road and amplify reach and impact. Moreover, such data can capture unintended results, alternative causes for outcomes, and multiple pathways to impact, all of which help explain why change is or is not happening.

#### Evaluate results and replan to improve and scale

The rigorous evaluation of SBC-related projects supports the commitment to evidence-based practice and continual learning. SBC is often complex, with many steps along the causal pathway that intertwines with the contextual landscape. Further, as is often the case for NSA, SBC may be one of many approaches in an integrated or multisectoral project with multiple activities, partners, and actors. This complexity can make it challenging to distinguish when a failure to detect an effect is related to an ineffective approach or activity or due to some other contextual factor (e.g., drought, conflict, policy, etc.). Because clear attribution of effect is impossible in most cases, framing evaluation in terms of contribution is more likely to reflect a realistic appreciation of context and complexity. Undoubtedly, evaluation that provides information on why the results were (or were not) achieved is often even more valuable than the results themselves. In 2021, the Medical Research Council and the National Institute for Health Research updated guidance on the development and evaluation of complex projects and noted that evaluations “should take into account the complexity that arises both from the intervention’s components and from its interaction with the context in which it is being implemented” [251].

Randomized controlled trials have long been considered the gold standard to inform the nutrition evidence base; however, their utility, feasibility, and affordability in evaluating large-scale, complex, or multisectorial public health and nutrition projects have been questioned [50,54,244]. Further, the act of individual or cluster randomization carries with it ethical concerns related to justice and equity [54,[252], [253], [254]]. Alternative randomization approaches, including stepped-wedge designs, have gained ground in response to these limitations. However, when randomized or experimental designs are not feasible or acceptable, plausibility or adequacy designs may be considered sufficient, but only if implemented rigorously. (For a discussion of different evaluation designs and their application in nutrition, we refer readers to the review by Habicht et al. [255]).

Nutrition and NSA projects often measure their core dietary outcomes, such as food consumption patterns, diet diversity, and nutrient intakes. However, few projects identify and evaluate impacts on the behavioral determinants that lie along the pathways to these outcomes or the intersecting influence of the contextual landscape [14]. An exception to this is knowledge, which continues to be used as a core indicator despite its poor predictive power for dietary behavior [14,256]. These behavioral determinants are often uncovered in formative research and can differ substantially from one context to the next. However, examples may include attitudes, self-efficacy, motivations, values, skills, intentions, social norms, food environment, and food security. These behavioral determinants often form the core assumptions underlying pathways that link project activities to outcomes. Identifying and monitoring these is critical to understanding why SBC programming succeeds or fails and can aid in identifying what needs improvement and how to improve it [257,258].

Often, the changes we seek to measure in NSA evaluations are emergent, nonlinear, unpredictable, and complex, making attribution impossible. Moreover, traditional reliance on quantification and correlational analysis “not only fails to capture the complexity of SBC processes, but actually distorts them” [244]. Complexity-aware methods allow us to get one step closer to appropriately accounting for the true nature of change. For example, contribution analysis allows teams to analyze the assumptions underpinning the project’s TOC to determine whether the SBC activities contributed to observed changes. If the assumptions underlying the TOC “hold up” to rigorous examination, then the argument for contributions is stronger [259]. However, the rigor of contribution analysis is only as strong as the TOC upon which it is based and the quality of the data collected. A poorly articulated TOC with minimally developed underlying assumptions or limited data on assumptions will yield a similarly weak contribution analysis. Other complexity-aware approaches include a diverse array of systems mapping and visualization (e.g., social network analysis, community mapping), modeling (causal loop diagrams), and narrative (Most Significant Change Technique, outcome harvesting) approaches [245]. The Core Group and others have developed toolkits to support implementers with thinking through SBC interventions and indicators while applying a complexity-aware lens to monitoring, learning, and evaluation [243,258,260].

When designed with complexity in mind, process and outcomes monitoring and impact evaluation can inform our understanding of whether SBC activities contribute to better outcomes. More importantly, particularly for scale-up and sustainability, they can elucidate how different components of the project work and how these components interact with each other and within a given context to contribute to broader impacts and system changes over time and at what costs [39,235,261]. Participatory mixed methods evaluation approaches, capture critical information to not only understand effectiveness but also inform decision making for implementation, sustainability, and scale-up. As noted by Skivington et al. [251], this broadening of evaluation goals “implies a shift from an exclusive focus on obtaining unbiased estimates of effectiveness towards prioritizing the usefulness of information for decision making”.

## Discussion

Dietary behaviors sit at the intersection of dynamic and context-dependent ecosystems comprising environmental drivers of food choices, such as agricultural production and food systems; community and household-related factors such as social norms, food culture, and food security; and individualized preferences, habits, and values [262]. NSA is increasingly seen as a useful strategy to facilitate change in this complex ecosystem as it addresses multiple immediate and underlying determinants of nutrition in LMICs. The design, implementation, and evaluation of effective SBC strategies to achieve dietary behavior change is paramount to their success.

**FIGURE 4:**
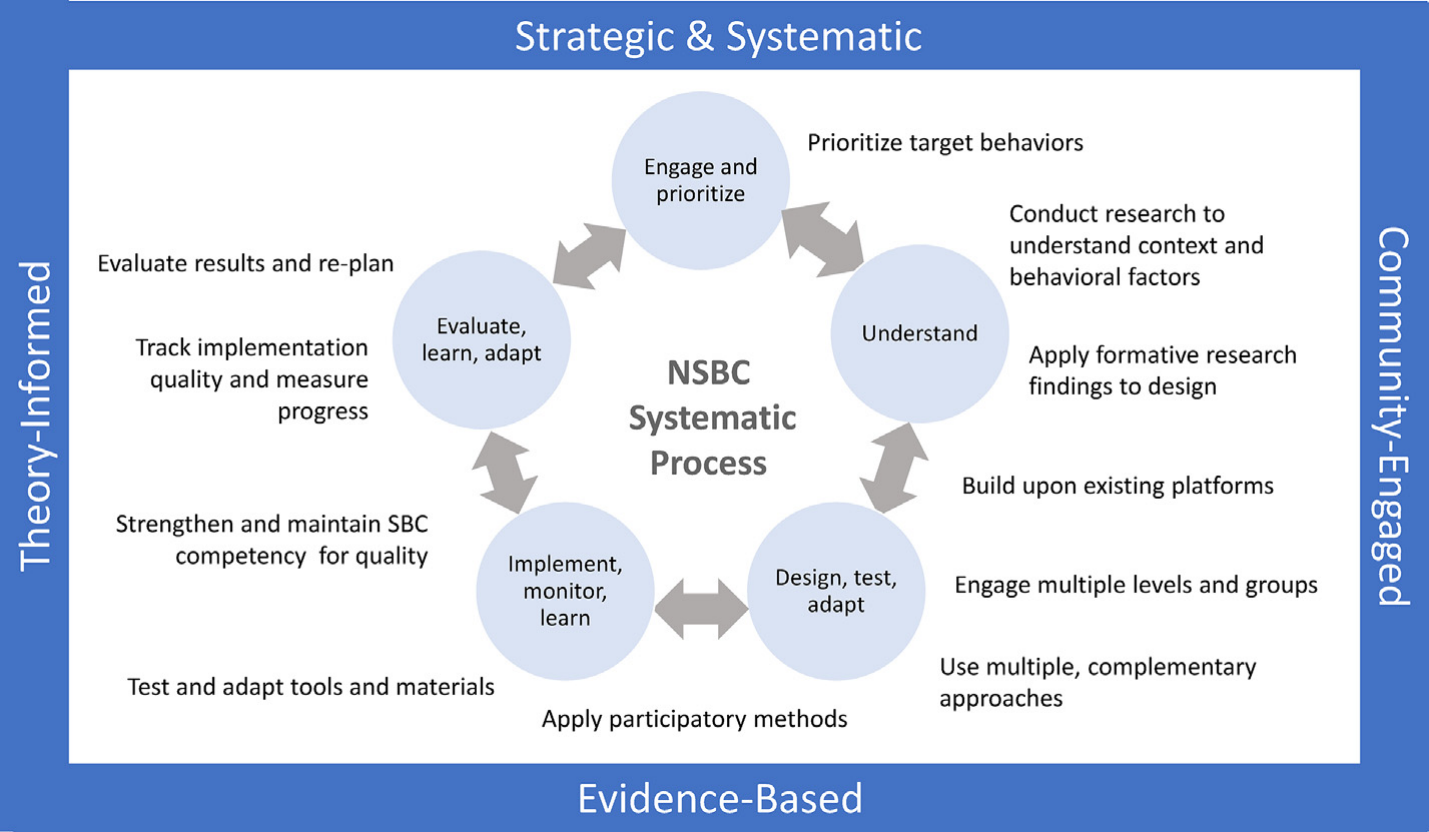
Core Principles and Practices for Designing, Implementing, and Evaluating Nutrition-Sensitive Agriculture Social and Behavior Change.

Through our narrative review of the peer-reviewed and gray literature, we identified 4 core principles and 11 core practices for the SBC process. We mapped these to a generalized 5-step SBC process based on the original P Process SBC framework and later iterations of a 5-step process (Supplemental Table 3). Our narrative review drew on a body of research and practice that reflects a broad consensus on those core practices and principles required for effective SBC. Although our narrative review focused more broadly on the field of nutrition in LMIC contexts, we also sought to identify and present specific examples and applications for NSA. We developed the framework graphic in Figure 4 to summarize these principles and practices. The 4 blue banners are the cross-cutting core principles that underlie the entire design, implementation, and evaluation process. The light blue circles capture the 5 steps in a systematic SBC process outlined in the first core principle. The black text represents the core practices, positioned relative to the steps in which they are commonly, though not exclusively, applied. Bidirectional arrows indicate the iterative nature of this process and reflect how, in a learning-focused approach, teams should revisit steps to adapt and improve the SBC strategy. Box 3 [[263], [264], [265], [266], [267], [268], [269], [270]] illustrates how one organization has applied multiple core principles and practices to the development and implementation of SBC in their NSA projects. To aid in the application of these core principles and practices, we have provided references organized by each principle and practice in Supplemental Table 2 and identified online, free guidance tools for implementers (Box 4 [23,28,34,35,[271], [272], [273], [274], [275], [276]]).

BOX 3 **A case study in applying multiple core social and behavior change principles and practices to nutrition-sensitive agriculture: The International Potato Center**

Two primary initiatives by the International Potato Center (CIP) to promote orange flesh sweetpotato (OFSP) – The *Sweetpotato for Profit and Health Initiative* (*SPHI*) and the *Sweetpotato Action for Security and Health in Africa* (*SASHA*) as well as CIP’s later work with OFSP promotion exemplify adherence with many of the core practices identified in this review [49,263,264]. Depending on formative research findings and identified behavioral determinants in each context, CIP’s OFSP projects collaborate with local stakeholders across diverse platforms to identify and implement a range of activities with the primary objective being to increase the production and consumption of OFSP, particularly among women and children. Activities are contextually adapted and may include, for example, farmers’ groups, women’s empowerment, schools, faith-based groups, engagement of husbands, and media outlets. Many of these serve as vehicles to introduce new techniques for growing OFSP and promoting OFSP consumption, for example social marketing strategies, cooking demonstrations, recipe competitions, and taste testing with OFSP dishes. Policy advocacy at local, regional, and national levels build buy in at local and country level. Market strategies simultaneously function to increase availability of OFSP through collaborations with private sector food industry partners such as food processors, bakers, and restaurants. Meanwhile, frontline workers such as community health workers or agricultural extension agents conduct home visits with families to provide more personalized support for dietary behavior change. Market-based advertising, radio spots, and community dialogs with elders, teachers, or influential farmers are utilized to shift social norms on gender and diet. In some contexts, CIP advocated for integrating OFSP into school feeding programs [265,266]. SBC strategies and materials are extensively field tested prior to larger roll out and throughout, as CIP recognizes the need for farmers to balance risk mitigation, productivity, and income with health [49,263,264]. In this way, projects layer mutually reinforcing and strategically designed approaches, including interpersonal communication, group education, media, community engagement, and advocacy across multiple levels. And they have done this work at scale. As of 2019, CIP’s OFSP promotion activities, as part of the Sweetpotato for Profit and Health Initiative, had reached 6.2 million households in 15 countries [49,263,264]. Depending on the aim, design, and context, CIP OFSP projects have significantly improved consumption of vitamin A-rich foods, diet diversity indicators, vitamin A intakes, vitamin A status, infant and young child feeding practices, and reduced child diarrhea [264,[267], [268], [269], [270]].

Alt-text: BOX 3

BOX 4 **Online Social Behavior Change Strategic Process Guidance Tools**

Behavior Centered Programming Training: An Approach to Effective Behavior Change [271]

Behavior Centered Design [272]

Building an SBC Strategy [273]

C-Change: C-Modules [23]

How to Build Your Theory of Change and Results Framework [274]

Social and Behavior Change in Nutrition: What Works? [275]

The FOCUS Tool- An SBC Planner [276]

The P Process [35]

The Behaviour Change Wheel [34]

Theory of Change Workbook: A Step-by-Step Process for Developing or Strengthening Theories of Change [274]

Using Research to Design a Social and Behavior Change Strategy for Multi-Sectoral Nutrition [28]

Alt-text: BOX 4

Our narrative review represents an iterative process informed by expert review, and the peer-reviewed and gray literature. Our work builds on prior reviews of the literature in the fields of SBC, maternal and child nutrition and NSA. These reviews largely established that SBC interventions, including when defined as nutrition education, can improve maternal and child diets and may be required for NSA interventions to achieve diet impacts [1,[5], [6], [7], [8], [9], [10], [11], [12],^14^,^43^,[277], [278], [279], [280]]. A limited number of reviews have also endeavored to identify specific behavior change techniques—defined as the smallest identifiable components with potential to change behavior [281,282]—that may contribute to changes in diet behavior. These reviews, including several on obesity interventions in high-income countries [[282], [283], [284], [285], [286]] and one analysis on complementary feeding in LMICs [17] applied a taxonomy of 93 specific behavior change techniques [287,288] to code specific techniques and identify which had stronger linkages to effectiveness. In contrast to these prior reviews, our narrative review does not seek to establish effectiveness of specific types of SBC interventions for nutrition but rather focuses on the *process* of SBC and what may be required to ensure that SBC is implemented with high quality to achieve anticipated impacts, particularly in the field of NSA. Our work also builds on the prior work of organizations and institutions to develop practice-based guidelines, toolkits, and resources for SBC. However, many of these utilized internal organizational learnings and experiences as opposed to providing a more comprehensive review of the peer-reviewed and gray literature.

These core practices and principles include reminders of the importance of structural and social factors and of engaging communities in a change process. However, over the course of this narrative review, we found the current status quo in SBC theory and practice remains weak on the “social” component of SBC. Global health and nutrition work is still predominantly focused on changing individual behaviors instead of social norms, drawing primarily on behavioral science instead of social systems theory, and emphasizing information-based rather than dialogical approaches [163,289,290]. These trends suggest that nutrition SBC, despite the complex and multicausal nature of nutrition [85] and an increasing focus in recent years on “co-creation” [291,292], may insufficiently draw on theories and methods from disciplines such as community development, anthropology, sociology, and community psychology [137,138,289,290]. As a result, the field of nutrition may struggle to operationalize SBC as an iterative, socioculturally grounded process of community-driven collective action for sustainable social change [85,291,292]. Developing, documenting, and measuring the impact of more culturally grounded approaches to investigating and improving dietary practices, as advocated by some, may facilitate further refinement of these core principles and practices [66,85,92,126,289,293].

### Strengths and limitations

We acknowledge that the core practices and principles outlined in this article do not cover all potentially valuable principles and practices; we limited the number of principles and practices to those meeting a high standard of broad applicability for SBC as it applies to nutrition in LMICs with emphasis on NSA. We excluded those that may only be pertinent to more specific activities (e.g., micronutrient supplementation) or that may be subsumed into other core principles or practices. For example, multisectoral integration is a critical practice for many NSA projects, as dietary practices are intertwined with factors in the health, environment, agriculture, and economic sectors. Coordinated implementation of complementary activities across sectors can bring synergistic results to projects. However, if the core practices of formative research, designing SBC activities to address underlying behavioral determinants, and working at multiple levels are applied, projects that would benefit from multisectoral integration would recognize this need through those practices and act accordingly within their resource constraints. (For those practitioners for whom multisectoral integration would be beneficial, there are several excellent reviews and case studies [3,168,[294], [295], [296], [297]]).

Additionally, we acknowledge gender equity and women’s empowerment is seen as a fundamental, cross-cutting issue requiring attention in diet, agriculture, and nutrition projects. Indeed, it is identified as a core pathway in most agriculture to nutrition frameworks. However, we did not include gender as a specific core practice or principle because the complex dynamics of gender are inherent in any sociocultural context. Relevant issues should be identified through the rigorous formative research process. Addressing discriminatory social norms and gender-based barriers and gaps through gender-transformative programming [141,298] would occur in the process of following other core practices in the design of strategically tailored, participatory, multilayered activities engaging women, girls, men, and boys in project settings. (There exists excellent reviews, case studies, and toolkits to support practitioners in gender transformative programming within nutrition interventions more broadly [[141], [142], [143],[299], [300], [301], [302], [303], [304], [305]] and in NSA interventions particularly [143,302]).

Although we were able to seek external review and inputs, we acknowledge that a more formal and participatory Delphi process to develop the framework based on more significant inputs was not possible at the time of framework development and represents a significant limitation. However, it is important to note that this document includes an initial framework to establish core practices within the field of SBC in NSA. This framework could and probably should be viewed as a living tool that changes as the field of SBC advances. The authors also acknowledge our positionality in this work as United States-based and educated practitioners whose SBC experience is often from the perspective of technical advisors and academic researchers working with in-country teams in LMICs. However, to reduce positionality bias, this work was reviewed by others throughout the review and development process, including a multidisciplinary panel of experts and participants at the global Agriculture, Nutrition and Health Academy annual conference in 2023.

Systematic reviews and meta-analyses provide a higher quality of evidence than a narrative review. However, the topic of this review was broad. Furthermore, it was not focused on the effectiveness or efficacy of a given intervention but rather a process that is highly heterogenous in its design, implementation, and evaluation. As a result, our team decided that a narrative review would be more appropriate and feasible. We recognize the biases inherent in a narrative review, including selection bias and author confirmation bias [31]. We attempted to minimize these biases through an iterative, team-based approach to literature and resource identification, discussion, and critique. We utilized broad search terms to cast a wide net and refined search terms in an iterative manner as findings emerged from the review. We also employed snowball and reverse snowball approaches for peer-reviewed and gray literature searches. We incorporated 2 rounds of external review by 2 different groups of experts who further assisted in refining our search strategies, identifying additional resources, reducing our own confirmation biases, and providing assurance that we had sufficiently explicated important core principles and practices. In the end, we consider this review as a starting point for greater discussion, debate, and research on the design, implementation, and evaluation of SBC in the field of nutrition and NSA. Ultimately, we recognize a more in-depth review or a series of systematic reviews focused on each of the core principles and practices would further advance, articulate, and refine this framework.

### Recommendations and next steps

To further the refinement and application of the core principles and practices, we recommend actions on the part of different key actors in the field of nutrition and NSA SBC. These recommendations highlight the need for continued investment in open access research, community engagement, transparency, and accountability to create a culture of sharing and learning.

#### Implementers, governmental, and nongovernmental actors

• Use this article as a resource to ensure core practices and principles are applied throughout each phase of the program cycle.
• Foster enhanced and transparent reporting that embodies a culture of learning. Go beyond sharing results to providing details on design, implementation, activities, processes, successes, AND failures.
• Prioritize community and participant-engaged methods for deeper engagement, co-design and shared learning.

#### Researchers

• Co-design studies together with people with technical expertise, as well as those with lived expertise, organizing expertise, and community history expertise who can contribute to implementation science on core practices and principles in nutrition SBC.
• Design, trial, and document innovative evaluation approaches that better capture the complexity of SBC interventions, particularly in multisectoral interventions.
• Conduct research that tests the value of individual core principles and practices to further understanding of what works and what does not work in the field of nutrition SBC.
• Investigate practitioner experiences with core practices such as behavioral prioritization, participatory engagement, and SBC capacity strengthening.
• With the generation of new evidence, continually revisit, update, and revise these core practices and principles.

#### Donors

• Ensure implementers are awarded sufficient funding, time, technical support, and other resources needed to enable the application of these principles and practices and sharing of experiences on their utilization.
• Require that funding proposals demonstrate how implementers will apply core principles and practices.
• Invest resources in capacity strengthening to support local partners to design, implement, monitor, and evaluate programs in alignment with the core principles and practices.

### Conclusions

Through a narrative review process, we have identified an explicit framework of core principles and practices for SBC for nutrition and NSA programming. Our goal is that this framework *1*) serves as a guide for design, research, implementation, and evaluation of such programs; *2*) helps standardize knowledge sharing and production; and *3*) contributes to improved quality of implementation. The development of this framework of core principles and practices is a necessary first step in what we hope will be a robust and collaborative process to articulate, justify, and codify core practices for SBC in the field of nutrition more broadly and NSA more specifically. The next steps for this work require research to validate these core principles and practices in partnership with program designers, implementers, and SBC experts in LMICs. As practitioners, researchers, and donors apply this framework to their work, new learnings, successes, and failures will emerge, allowing continual refinement. As such, we welcome feedback from readers to facilitate an iterative process of review, learning and application, and adaptation.

## Author contributions

The authors’ contributions were as follows – MP-W, AWG: developed the initial concept for the article; MP-W, LG, TT, EF, AWG: contributed to drafting core principles and practices, conducted the narrative review, drafted sections of the manuscript, contributed to revisions; MP-W, LG, AWG: have primary responsibility for final content; and all authors: read and approved the final manuscript.

## Conflicts of interest

The authors report no conflicts of interest.

## Funding

This work was funded by the Bill and Melinda Gates Foundation. The funder had no involvement in or restrictions on the published work.
