# Supplementary material for: Core Principles and Practices for the Design, Implementation, and Evaluation of Social and Behavior Change for Nutrition in Low- and Middle-Income Contexts with Special Applications for Nutrition-Sensitive Agriculture
Source: Curr Dev Nutr. 2024 Jul 14;8(8):104414. doi: 10.1016/j.cdnut.2024.104414 (PMC11367532; doi:10.1016/j.cdnut.2024.104414)
Supplement: multimedia component [file mmc1.docx]

| **Supplemental Table 1: Links to the Social and Behavior Change Guidance Sources Reviewed to Inform Preliminary Draft of Core Principles and Practices** |
| --- |
| Guidance Documents and Toolkits   - [Social and Behaviour Change (SBC) for Improved Agriculture and Nutrition, Webinar Series](https://www.anh-academy.org/anh-academy/partnerships/giz-social-behaviour-change) (2020). Agriculture, Nutrition and Health Academy in partnership with GIZ - FHI360, C-Change: [C-Modules](https://www.fhi360.org/resources/c-modules-learning-package-social-and-behavior-change-communication/) and C-Bulletins - Health Communication Capacity Collaborative, Johns Hopkins University - [The P-Process](https://www.healthcommcapacity.org/wp-content/uploads/2014/04/P-Process-Brochure.pdf) - GIZ [- Social and Behavior Change: Insights and Practice](https://www.behaviourchange.net/document/240-social-and-behaviour-change-insights-and-practice) - Ideo - [The Field Guide to Human-Centered Design](https://www.designkit.org/) - The Manoff Group - [Think/BIG: Behavior Integration Guidance](https://thinkbigonline.org/index) - Core Group/USAID - [Make Me a Change Agent: An SBC Resource for WASH, Agriculture, and Livelihoods Activities](https://www.fsnnetwork.org/mmca) - [Save the Children - FOCUS Tool: An SBC Planner](https://www.healthynewbornnetwork.org/resource/the-focus-tool/) - RANAS-MOSLER - [Behavior Change Manual for WASH Projects: The RANAS Approac](https://www.fsnnetwork.org/resource/behavior-change-manual-wash-projects)h; [The RANAS approach to behavior change](https://www.eawag.ch/fileadmin/Domain1/Abteilungen/ess/projekte/EHPsy/Methodological_Fact_Sheets.pdf) - [CORE Group - Designing for Behavior Change Guides](https://coregroup.org/resource-library/designing-for-behavior-change-for-agriculture-natural-resource-management-health-and-nutrition/) - CORE Group - [Make me a Change Agent: A Multisectoral SBC Resource](https://coregroup.org/wp-content/uploads/media-backup/documents/Resources/Tools/MMCA_English_Final.pdf) - UNICEF - [Behavioral Drivers Model](https://www.unicef.org/mena/reports/behavioural-drivers-model) - World Bank [- FOAM Framework](https://elibrary.worldbank.org/doi/abs/10.1596/27924) (Focus on Opportunity, Ability, and Motivation) - Michie, S., Atkins, L. West, R. 2014. [The Behavior Change Wheel](https://www.behaviourchangewheel.com/). Silverback Publishing   Various webinars, technical and training materials from:   - USAID’s [SPRING](https://spring-nutrition.org/) and [Advancing Nutrition](https://www.advancingnutrition.org/sites/default/files/2020-05/defining_social_and_behavior_change_brief.pdf) Programs - [Alive and Thrive](https://www.aliveandthrive.org/en) - USAID and Johns Hopkins University. [Breakthrough ACTION & RESEARCH](https://breakthroughactionandresearch.org/),   Multiple resources from online resource hubs:   - [Springboard for Social and Behavior Change](https://springboardforsbc.org/) - [Health Communication Capacity Collaborative](https://healthcommcapacity.org/) (HC3) - [The Compass for SBC](https://thecompassforsbc.org/)   Report and Strategic Agenda for Nutrition SBCC and multiple presentations from the 2014 conference: [Designing the Future of Nutrition Social and Behavior Change Communication](https://www.advancingnutrition.org/sites/default/files/2022-05/spring_gain_sbcc_conference_strategic_agenda_report.pdf) |

| Supplemental Table 3. Detailed overview of various processes for planning strategic, evidence-based SBC programs | | | |
| --- | --- | --- | --- |
| P Process, 2013 (1) | **C-Change, 2012 (2)** | **Behavior Centered Design, 2017 (3)** | **USAID Advancing Nutrition, 2020 (4)** |
|  | | **Prioritize behaviors**   1. Determine nutritional status or note the nutrition-sensitive outcome 2. For each of the relevant behaviors, analyze the behavior gap, potential to impact results, and potential ability to change 3. Narrow the behaviors of interest by determining program and policy fit 4. Select final prioritized behaviors | |
| Inquire   1. Conduct baseline and/or formative research and review literature 2. Identify audiences 3. Uncover intended audiences’ barriers to behavior change (economic, social, structural, cultural or educational, etc.) 4. Identify facilitating factors for behavior change, including potential messengers and media 5. Develop problem statement | **Understanding the situation**   1. Develop problem tree 2. Conduct people analysis 3. Conduct context analysis (includes gender perspective) 4. List existing research inventory 5. Develop research plan 6. List partners, allies, and gatekeepers 7. Develop summary of analyses 8. Develop the ToC | **Assess**   1. Review the literature speak to experts 2. Define the behaviors that need to change, what is known about the determinants of the behavior(s) (using the BCD checklist), the target audience, the context for the intervention 3. Facilitate framing workshop to review what is known, develop the ToC, and decide what questions still need to be answered | **Plan and Conduct Research**   1. Conduct literature review on the main barriers and enablers or "factors" that influence someone to practice and maintain prioritized behaviors. 2. Plan formative research (if needed) 3. Conduct formative research (if needed) |
| Design Strategy   1. Develop communication objective(s) 2. Conduct Audience segmentation 3. Identify approaches and positioning 4. Identify communication channels 5. Develop implementation plan 6. Develop monitoring and evaluation plan 7. Develop dissemination plan | **Focusing & Designing**   1. Develop communication strategy 2. Conduct audience segmentation 3. Conduct audience prioritization 4. Conduct audience profile 5. Develop SMART communication objectives 6. Develop strategic approach 7. Identify positioning for social and behavior change communication (SBCC) materials 8. Develop activity, channel, and material mix plan 9. Develop implementation plan | **Build**   1. Conduct formative research 2. Use BCD checklist to organize findings 3. Identify insights 4. Develop creative brief | **Design a Nutrition Social and Behavior Change Strategy**   1. Analyze research findings 2. Confirm or refine priority behaviors 3. Highlight or star factors and actors most closely related to the refined behaviors 4. Create linked pathways from SBC determinants to activities 5. Develop the SBC strategy (Outlines priority behaviors, summarizes the analysis, describes the priority groups and supporting actors, discusses the key factors, lists the activities and includes implementation, monitoring, and evaluation and learning plans) |
| Create & Test   1. Develop communication products 2. Test ideas and designs with intended audiences 3. Revise, retest and finalize communication products | **Creating**   1. Inventory existing materials and activities 2. Analyze examples of SBCC materials 3. Develop creative brief(s) for SBCC materials 4. Draft production timeline 5. Draft SBCC materials | **Create**   1. Design intervention strategies and materials 2. Develop prototypes of intervention materials and touchpoints 3. Test prototypes 4. Make adjustments to the prototypes and test until ‘matured’ 5. Present intervention package to the implementing team and other stakeholders | **Plan for Implementation and Monitoring, Evaluation, and Learning**   1. Define SBC competencies (and CHW competencies if relevant) to guide hiring decisions, identify areas for capacity strengthening, and track changes in performance over time 2. Identify appropriate indicators (See People in Need’s Indikit- <https://www.indikit.net/>) 3. Selecting and applying methods 4. Analyze results 5. Make adaptations |
| Mobilize & Monitor   1. Implement and monitor progress 2. Make mid-course corrections 3. Prepare for further evaluation activities | **Implementing & Monitoring**   1. Develop detailed workplan 2. Develop project staffing plan 3. Identify SBC coordination and supervisory mechanisms 4. Develop budget / budget priorities concerning SBCC 5. Develop sequence, timing, and synergy plan 6. Develop tracking plan for distribution points and production needs (per material or activity) 7. Develop plan to monitor process and quality of SBCC materials and activities 8. Identify gender issues related to planning, implementation, and evaluation 9. Identify and approach resource providers | **Deliver**   1. Implement the intervention 2. Use the ToC to identify critical points that could affect implementation 3. Monitor delivery of interventions for exposure, dose, coverage, fidelity, acceptability, simplicity, brand recognition, evaluability, sustainability and security | **Implement, Monitor, and Adapt**   1. Monitor and adjust programming as needed in order to improve quality |
| Evaluate & Evolve   1. Measure outcomes and assess impact 2. Disseminate results 3. Develop final report | **Evaluating & Re-planning**   1. Select M&E questions 2. Select monitoring and evaluation indicators 3. Develop M&E plan 4. Design M&E methods and tools 5. Develop data analysis plan 6. Conduct re-planning exercise | **Evaluate**   1. Gather evidence of outcomes and impact- whether the expected cause—effect linkages in the ToC actually occurred 2. Gather process data to test for active psychological, social and physical environmental changes associated with programming 3. If financially possible, measure the sustainability of the behavior change | **Evaluate**   1. Use the MEL plan to guide the design of the evaluation of SBC approaches |
| Citations   1. Health Communication Capacity Collaborative. The P Process. Five Steps to Strategic Communication. Baltimore: Johns Hopkins Bloomber School of Public Health Center for Communication Programs, 2013. Available at <https://thecompassforsbc.org/wp-content/uploads/P20Process20Eng202620Fr.pdf> 2. C-Change. C­Modules: A Learning Package for Social and Behavior Change Communication (SBCC). Washington, DC: C-Change / FHI 360, 2012. Available at <https://www.fhi360.org/sites/default/files/media/documents/Cover.pdf> 3. Aunger, R., White, S., Greenland, K., Curtis., V. Behavior Centered Design: A Practitioner’s Manual. London School of Hygiene and Tropical Medicine. Version 1. February 2017. Available at <https://www.lshtm.ac.uk/sites/default/files/2017-03/BCD%20Manual.pdf> 4. USAID Advancing Nutrition. Using research to design a social and behavior change strategy for multi-sectoral nutrition. Arlington, VA: USAID Advancing Nutrition, 2021. Available at: <https://www.advancingnutrition.org/sites/default/files/2021-08/research_to_design-tool_for_high-quality_sbc.pdf> | | | |

| **Supplemental Table 4**. Common social and behavior change approaches, their definitions and nutrition related examples. | | |
| --- | --- | --- |
| **Approach** | **Definitions of approaches** | **Examples of activities** |
| **Enabling Environment Approaches** | | |
| Advocacy | Communications and activities designed to raise awareness, shape policy, stir commitment to program objectives, and mobilize resources. | - Advocate with private sector to package nutrient-rich foods in smaller portion sizes. - Build partnerships with governments and media to support NSA programming and influence the public health agenda. |
| Social Mobilization | Activities engaging stakeholders, leaders, and communities to raise awareness, build buy-in for ideas, foster commitment to action, and mobilize resources; generally focused on broader socio-structural change. | - Build linkages between health providers and marginalized communities. - Mobilize community action to establish clinical and nutrition services in unreached areas. - Identify common interests with faith-based leaders and mobilize their influence to support key MIYCN practices in their communities. |
| Behavioral Economics | Approaches drawing on psychology and economics use changes (‘nudges’) in the physical and social environment to trigger behavioral choices that may be less conscious or rational. | - Strategically position nutritious foods in food service, education and advertising so prominence subtly guides choices. (Shaping the choice architecture). - Promote use of pre-marked serving dish to feed child appropriate foods. |
| Capacity Strengthening | Approaches designed to develop knowledge, skills, and motivation—for target population to practice the new behaviors, and for policy makers, program managers, and frontline workers to plan, design, and deliver SBC activities effectively. | - Train program staff in SBC principles and skills for facilitating community activities that support positive nutrition behaviors. - Provide ongoing coaching and mentoring to frontline workers and facilitate peer learning for improvement of skills. |
| Provision of material/inputs | Distribution of any type of material benefits to participants, with the aim to improve access and ability to practice the new behavior. | - Develop take-home materials that illustrate food groups and remind of nutrition guidance. - Provide egg-laying chickens, seeds and farm inputs for homestead gardening initiatives |
| **Community-Based Approaches** | | |
| Community Outreach | Generally involving dissemination of information or resources on a specific issue than mobilizing for social change. Often works through a particular agency, using 1-way education as well as IPC. | - Organize community-level education events, health screenings, and demonstrations to increase community awareness about the availability of nutrition services. |
| Community Engagement | Used in more varied ways, this is a broader term. Compared to “outreach,” “engagement” highlights community participation in behavior change efforts. It involves working with the community through ongoing relationships, often focused on organized groups. Uses participatory activities that aim to promote dialogue, joint decision-making, empowering action. | - Establish or strengthen local governance and oversight of community structures, such as community action groups and community health committees. - Integrate nutrition education and demonstrations in existing community groups (e.g. village savings and loans, youth club, farmers co-op). |
| Community Dialogue | Relies on skillful facilitation and applies principles of adult learning and transformational learning theory to drive systems-level change while developing capacity. Engages whole communities in a participatory process of critical reflection and decision making. Prioritizes listening to community voices and working with local cultural assets. | - Facilitate group exercises to explore social norms related to diets, using storytelling, debate, and discussion to explore varied reasons behind the lack of dietary diversity. - Facilitate collective exercises in nutrition sensitive agricultural planning. |
| Group Education | Approaches with a clear aim to teach information and/or skills, usually including interaction within groups. | - Present NSA-related information at schools, peer group meetings, and health events. |
| Community mobilization | Can include a wide range of community-based activities that aim to engage local leaders’ support and engage community members in social and behavior change, but with more emphasis on social change than specific behaviors compared to Community Engagement. Seeks broad participation across the community (as opposed to targeted groups), engaging multiple actors for an integrated approach and collective ownership. | - Develop nutrition champions among civic leaders. Support them to lead community sessions and promote participation in new initiatives. - Engage youth to develop peer-based campaigns using art, music and drama that promote healthy nutrition practices. |
| **Communication (BCC) approaches** | | |
| Interpersonal Communication (IPC) | Two-way, direct, personal communication, generally face-to-face. Includes one-on-one, or interactions between groups small enough for conversation (generally < 15 people). Conducted in homes, public spaces, health facilities, anywhere. | - Provide counseling during home visits with caregivers to support improved feeding practices. - Facilitate small peer group discussions to overcome challenges with new feeding practices. |
| Entertainment education (“edutainment”) | A communication strategy that promotes behavior or social change through entertainment performed live in community spaces or broadcast on mass media via TV, radio, or internet. Performance is designed to educate and entertain simultaneously. Generally one-way communication but can be combined with community-based approaches and individual or group discussion (IPC) for deeper impact. | - Conduct radio plays to promote the benefits of joint decision-making within households. - Organize youth to create and perform dramas showing the benefits of improved nutrition. |
| Social Marketing | The application of marketing methods and concepts to promote products, services or behaviors for health as a social good. Highlights the audience perspective and uses a mix of media to address needs and wants to achieve objectives. | - Conduct promotional campaign (using peer education, media, community influencers) to increase interest in purchasing nutrient-rich foods. |
| Mass Media | Broadcast and print media that reaches a wide audience at large scale. | - Produce radio programs dramatizing households implementing dietary changes. - Engage journalists to publish stories on NSA-related topics in local newspapers. - Engage local celebrities to perform songs at community fairs supporting program messages. |
| Mid-media | Media distributed through a small, defined, geographic area. | - Use community public address system and posters to promote NSA-related messages. - Hold sessions with women's groups to view NSA-related videos and discuss ways to overcome barriers to adopting recommended practices. |
| Small media | Media distributed to individuals and small groups. | - Distribute flyers, stickers or calendars with key messages and engaging visuals to teach and remind people of key messages. - Create counseling cards for use in sessions with caregivers. |
| Digital and Social media/ICT | Information and Communication Technologies using the internet and cellular phone networks. May have broad reach or be targeted to individualized interactions. | - Broadcast key messages through popular social media apps - Use SMS to support frontline workers with reminders and facilitate data collection. - Offer eLearning programs to implementing staff. |
| **Other approaches** | |  |
| Human-centered design (HCD) | An interdisciplinary problem-solving approach that focuses on eliciting the participants’ perspectives and mobilizing their participation to generate ideas creatively and test prototypes in a rapid, iterative, cost-effective fashion. Used for learning and design, it can be considered an implementation approach that fosters motivation for SBC among participants and helps projects mobilize tested, tailored behavior change solutions. | - Design labor rotating or pooling approaches with farmer groups. - Work with mother care groups to develop tools to guide appropriate young child feeding. |
